# Lignin-Derived Biochar in Biorefineries: Linking Structure–Property Relationships to Emerging Contaminant Removal and Controlled Release Applications

**DOI:** 10.3390/molecules31122116

**Published:** 2026-06-16

**Authors:** Francisco Flores-Céspedes, Luis García-Fuentes

**Affiliations:** Department of Chemistry and Physics, University of Almería, Crta. Sacramento s/n, 04120 Almería, Spain; lgarcia@ual.es

**Keywords:** lignin valorization, lignin-derived biochar, adsorption mechanisms, emerging contaminants, controlled release systems, sustainable agriculture, circular bioeconomy

## Abstract

Lignin is an abundant aromatic biopolymer generated as a major by-product in lignocellulosic biorefineries, and its efficient valorization is essential for improving process sustainability and economic viability. Among current upgrading strategies, the conversion of lignin into lignin-derived biochar (LDB) has emerged as a promising route because of its high carbon yield, scalable production, and tunable physicochemical properties. This review examines the relationships between lignin structure, thermochemical conversion pathways, and the resulting properties of LDB materials within biorefinery systems. The influence of different technical lignins and conversion routes, including pyrolysis and hydrothermal carbonization, is critically discussed together with post-functionalization strategies. Particular attention is devoted to emerging applications in contaminant adsorption and controlled release systems for agrochemicals. The adsorption mechanisms governing pharmaceuticals, pesticides, microplastics, and PFAS removal are analyzed, while the dual role of LDB as both adsorbent and delivery platform is highlighted. Current limitations include lignin heterogeneity, lack of standardized evaluation protocols, and insufficient validation under realistic environmental conditions. Overall, LDB represents a versatile and scalable platform for lignin valorization and sustainable material design within circular bioeconomy frameworks.

## 1. Introduction

Lignin, the most abundant renewable aromatic polymer, remains significantly underutilized in modern biorefineries. Despite its high carbon content and structural richness, it is still predominantly valorized through low-value energy recovery processes, such as combustion in pulping and bioethanol industries [1,2]. This underutilization contrasts sharply with its potential as a sustainable precursor for high-value chemicals and functional carbon materials.

**Figure 1:**
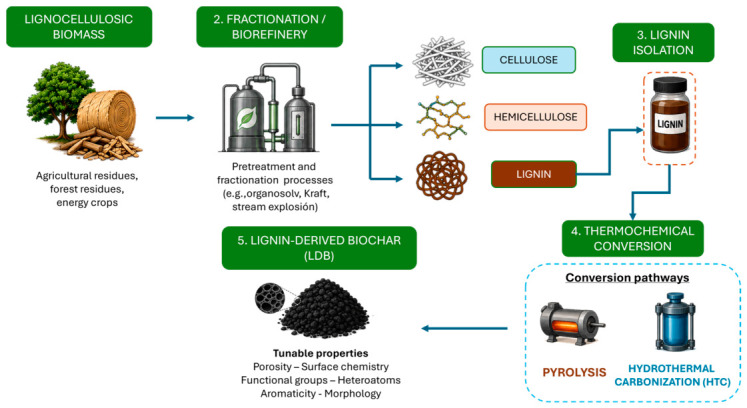
Schematic representation of the production of lignin-derived biochar (LDB) from lignocellulosic biomass through biorefinery fractionation, lignin isolation, and thermochemical conversion processes.

**Figure 2:**
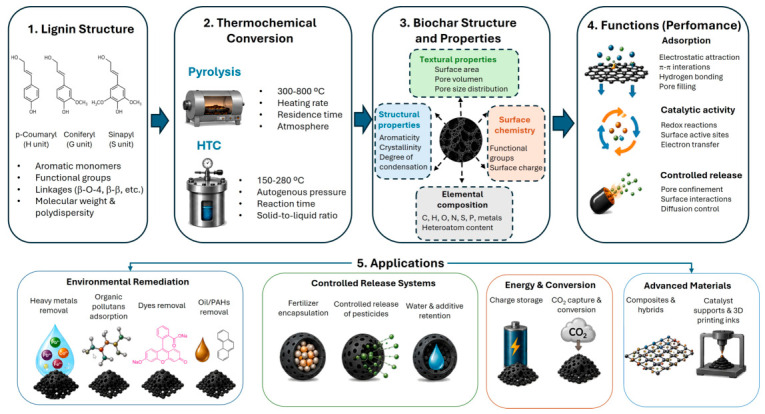
Structure–property–function framework of lignin-derived biochar, linking lignin structure, thermochemical conversion processes, material properties, and their applications.

In recent years, increasing attention has been devoted to lignin valorization as a key strategy for improving the economic viability of lignocellulosic biorefineries [3,4]. Within this context, the conversion of lignin into carbon-based materials has emerged as a particularly promising pathway. Among these materials, lignin-derived biochar (LDB) is particularly attractive due to its relatively simple production, high carbon yield, and tunable physicochemical properties [5,6,7,8]. Through thermochemical processes such as pyrolysis or hydrothermal carbonization, lignin can be transformed into porous carbon materials with controlled surface area, pore structure, and surface functionality [9,10]. The overall lignin valorization pathway within biorefineries and its integration into material applications are schematically illustrated in Figure 1, while the structure–property–function relationships governing lignin-derived biochar are summarized in Figure 2.

The properties of lignin-derived biochar are strongly influenced by both the molecular structure of lignin and the conditions of thermochemical conversion. Compared with biochars derived from cellulose- or hemicellulose-rich feedstocks, lignin-based biochars typically exhibit higher aromaticity, greater structural stability, and enhanced adsorption capacity [11,12]. These characteristics make LDB particularly attractive for applications in environmental remediation and advanced material systems [13].

In environmental applications, lignin-derived biochar has demonstrated considerable potential for the removal of emerging contaminants from water and soil systems, including pharmaceuticals, antibiotics, pesticides, and per- and polyfluoroalkyl substances (PFAS) [14,15,16,17,18]. However, the reported performance is often highly dependent on activation conditions and remains difficult to compare across studies. This performance is governed by multiple interaction mechanisms, including π–π interactions, hydrogen bonding, electrostatic attraction, and pore-filling effects, which are closely linked to the aromatic carbon framework and surface functional groups of LDB [10,19].

In parallel, LDB has recently emerged as a promising platform for controlled release systems [20,21]. Its porous structure enables the adsorption and gradual release of active compounds such as fertilizers, micronutrients, and pesticides, thereby contributing to improved resource use efficiency and reduced environmental losses. Although controlled release systems based on lignin and biochar have been increasingly explored, the specific role of lignin-derived biochar in these systems remains insufficiently elucidated.

Despite these advances, several critical challenges remain. In particular, the intrinsic heterogeneity of lignin, the variability of technical lignin feedstocks, and the limited understanding of structure––property–function relationships hinder the rational design of lignin-derived biochar materials. Furthermore, although lignin-derived carbons have been increasingly investigated, a substantial proportion of the literature still focuses on biochars produced from whole lignocellulosic biomass or mixed feedstocks, while comparatively fewer studies systematically isolate the role of purified technical lignins as standalone precursors.

Within the broader context of lignocellulosic biorefineries, lignin-derived biochar offers a complementary valorization pathway compared to catalytic depolymerization or biochemical conversion routes. While these alternative strategies aim to produce defined chemical intermediates, biochar production provides a robust and scalable approach that tolerates lignin heterogeneity and requires less stringent purification. This positions LDB as a particularly attractive option for integrating lignin streams into value-added material applications, especially in facilities where process simplicity and carbon efficiency are prioritized.

In this context, the present review provides a comprehensive and critical overview of lignin-derived biochar within the framework of modern biorefineries. Particularly focused on (i) the structure and sources of lignin, (ii) thermochemical conversion pathways for LDB production, (iii) physicochemical properties and functionalization strategies, and (iv) emerging applications in environmental remediation and controlled release systems. Emphasis is placed on elucidating structure–property–function relationships and identifying future research directions for the development of lignin-based functional materials within sustainable and circular bioeconomy systems. As outlined in Figure 1, this review follows the conceptual pathway from lignin structure and conversion to functional material applications, providing a unified framework for understanding LDB within biorefineries.

## 2. Structure and Properties of Lignin

Lignin is a highly complex and heterogeneous biopolymer that constitutes one of the main structural components of lignocellulosic biomass, typically accounting for approximately 15–40% of its dry weight. It plays a fundamental role in plant cell walls by providing mechanical strength, hydrophobicity, and resistance to enzymatic and microbial degradation [22,23,24]. Due to its abundance and high carbon content, lignin represents the largest renewable source of aromatic structures in nature, making it an especially attractive feedstock for the development of carbon-based materials within modern biorefineries.

Chemically, lignin is formed through the radical polymerization of phenylpropanoid units derived from three primary monolignols: p-coumaryl alcohol, coniferyl alcohol, and sinapyl alcohol. These monolignols give rise to p-hydroxyphenyl (H), guaiacyl (G), and syringyl (S) structural units, respectively [22,24,25]. The resulting polymer exhibits a highly irregular three-dimensional network in which these units are interconnected through a variety of ether and carbon-carbon linkages, including β-O-4, β-β, β-5, and 5–5 bonds. Among these, β-O-4 linkages are typically the most abundant and play a key role in determining lignin reactivity during thermochemical conversion.

The relative abundance of H, G, and S units varies significantly depending on the biomass source. Softwoods are generally rich in guaiacyl units, hardwoods contain both guaiacyl and syringyl units, and grasses exhibit a more heterogeneous composition that includes all three types. These compositional differences directly influence lignin reactivity, thermal stability, and fragmentation pathways during conversion processes, which in turn affect the properties of lignin-derived materials.

The relative abundance of S, G, and H units significantly influences lignin behavior during thermochemical conversion. Guaiacyl-rich lignins generally promote the formation of more condensed aromatic structures because the free C5 position facilitates additional carbon–carbon coupling reactions during pyrolysis. In contrast, syringyl-rich lignins, which contain two methoxy groups, exhibit lower crosslinking density and may generate less condensed carbon frameworks. H-type units are typically associated with higher thermal reactivity and faster fragmentation pathways. Consequently, the S/G/H ratio directly affects carbon yield, aromatic condensation, pore development, and surface functionality in lignin-derived biochar [26].

In addition to its natural variability, lignin structure is further modified during industrial processing. Different extraction methods produce distinct types of technical lignin, including kraft lignin, lignosulfonates, organosolv lignin, and soda lignin. These lignins differ in molecular weight distribution, sulfur content, degree of condensation, and functional group composition, all of which significantly influence their behavior during thermochemical conversion and their suitability for specific valorization pathways [1,27].

From a materials science perspective, the structural complexity of lignin represents both a challenge and an opportunity. On the one hand, its heterogeneity and irregular architecture hinder the establishment of predictable and reproducible conversion pathways, thereby complicating process optimization. On the other hand, the presence of a wide variety of functional groups, including phenolic hydroxyl, aliphatic hydroxyl, methoxy, and carbonyl moieties, provides multiple reactive sites that can be exploited for chemical modification and interaction with target molecules [28].

These structural characteristics are particularly relevant to the production of lignin-derived biochar. During thermochemical conversion processes such as pyrolysis or hydrothermal carbonization, lignin undergoes dehydration, cleavage of ether linkages, and condensation of aromatic fragments, leading to the formation of carbon-rich materials with highly aromatic domains. The initial composition of lignin, including the S/G ratio and the distribution of interunit linkages, plays a key role in determining carbon yield, the degree of aromatic condensation, and pore development in the resulting biochar [9,29].

Biomass feedstocks with higher lignin content generally produce biochars with increased fixed carbon content, greater aromaticity, and enhanced structural stability compared with those derived from cellulose- or hemicellulose-rich materials. These properties are directly associated with improved adsorption capacity and long-term environmental stability [9,30,31]. In particular, the aromatic carbon framework inherited from lignin contributes to strong interactions with organic molecules, which is essential for applications in contaminant removal and controlled release systems.

However, despite these advantages, the relationship between lignin structure and the physicochemical properties of lignin-derived biochar is not yet fully understood. The intrinsic heterogeneity of lignin, combined with the variability introduced during extraction and processing, makes it difficult to establish clear structure–property–function relationships. This represents a major bottleneck for the rational design of lignin-derived biochar and highlights the need for more systematic studies linking lignin chemistry to material performance.

Notably, most studies do not isolate the effect of lignin structure from processing conditions, which complicates the interpretation of the reported trends. These structural features ultimately dictate thermochemical conversion pathways, as discussed in the following section.

## 3. Lignin Conversion and Biochar Production

The conversion of lignin into value-added materials represents a key strategy for improving the economic viability of lignocellulosic biorefineries. Among the available valorization pathways, thermochemical processes such as pyrolysis and hydrothermal carbonization (HTC) have attracted considerable attention because of their ability to transform lignin into carbon-rich materials with tunable physicochemical properties. In particular, the production of lignin-derived biochar provides a direct route for converting lignin into functional carbon materials for environmental and agricultural applications.

The properties of lignin-derived biochar, including surface chemistry, pore structure, and adsorption capacity, are strongly influenced by both the characteristics of the lignin feedstock and the operating conditions employed during thermochemical conversion [30,32,33]. As discussed in Section 2, the aromatic structure, degree of condensation, and functional group distribution of lignin play a decisive role in determining its thermal behavior and the structure of the resulting biochar. Consequently, understanding the interplay between lignin chemistry and processing conditions is essential for controlling biochar properties [9].

### 3.1. Pyrolysis of Lignin

Pyrolysis is the most widely applied method for producing lignin-derived biochar. This process involves the thermal decomposition of lignin in the absence of oxygen, typically at temperatures ranging from 400 to 800 °C. During pyrolysis, lignin undergoes a series of complex reactions, including depolymerization, devolatilization, and condensation, which lead to the formation of solid carbonaceous residues together with liquid and gaseous by-products [34].

At relatively low temperatures, lignin decomposition is incomplete, resulting in biochars with higher oxygen content and less developed aromatic structures. As the temperature increases, the progressive loss of volatile compounds and enhanced carbonization promote the formation of more condensed aromatic networks. This results in biochars with higher fixed carbon content, increased hydrophobicity, and improved thermal stability [9].

Pyrolysis temperature also plays a critical role in pore development. Higher temperatures generally favor the formation of microporous structures and increase surface area, which in turn favor adsorption applications. However, these conditions may simultaneously reduce the abundance of oxygen-containing functional groups, potentially limiting interactions with polar molecules and affecting performance in applications where surface functionality is critical. Moreover, the trade-off between increased porosity and the loss of surface functionality is not systematically addressed in the literature, making it difficult to establish optimal pyrolysis conditions for specific applications [35,36,37,38].

In addition to temperature, other parameters such as heating rate, residence time, and reactor configuration influence the structural evolution of lignin-derived biochar. Slower heating rates may promote more uniform carbonization, whereas longer residence times can enhance structural ordering and aromatic condensation [39,40]. These parameters provide important tools for tailoring biochar properties to specific application requirements.

### 3.2. Hydrothermal Carbonization

Hydrothermal carbonization represents an alternative thermochemical route for converting lignin into biochar-like materials under milder conditions. This process is typically carried out in aqueous environments at temperatures between 180 and 250 °C under autogenous pressure. In contrast to pyrolysis, HTC involves a combination of hydrolysis, dehydration, and condensation reactions occurring in the liquid phase [41,42].

A key characteristic of HTC-derived materials is the retention of a higher fraction of oxygen-containing functional groups. Compared with pyrolyzed biochars, these materials generally exhibit higher surface polarity and different pore structures, which can enhance their interaction with polar compounds. This behavior can be advantageous for applications such as nutrient retention and controlled release systems [43,44].

However, HTC-derived biochars typically show lower aromaticity and reduced structural stability compared with those produced by high-temperature pyrolysis. As a result, their performance in adsorption applications may be limited, particularly for non-polar organic contaminants. Therefore, the choice between pyrolysis and hydrothermal carbonization should be guided by the specific requirements of the intended application. Overall, pyrolysis and hydrothermal carbonization offer complementary pathways for tailoring LDB properties: pyrolysis favors highly aromatic and stable carbon structures, whereas HTC preserves oxygen-containing functionalities and enhances surface polarity [42,43].

Although pyrolysis and HTC are the most widely studied routes for the production of lignin-derived biochar, other thermochemical conversion technologies have also been explored [45,46,47]. Torrefaction, typically conducted at 200–300 °C under inert conditions, can improve lignin carbonization and fuel properties, although it generally produces materials with lower porosity and surface area than conventional biochars. Gasification, performed at higher temperatures in the presence of controlled amounts of oxidizing agents, is primarily intended for syngas production and usually results in lower solid carbon yields. Consequently, these approaches have received comparatively less attention for the preparation of functional lignin-derived biochar materials. For this reason, the present review focuses on pyrolysis and hydrothermal carbonization, which constitute the dominant pathways reported in the literature for generating lignin-derived biochars with tailored properties for environmental and agricultural applications.

### 3.3. Process–Structure Relationships

From a materials design standpoint, the properties of lignin-derived biochar are governed by the combined effects of feedstock characteristics and thermochemical processing conditions. The selection of lignin type, together with the optimization of temperature, residence time, and reaction environment, determines key properties such as surface area, pore size distribution, degree of aromatic condensation, and surface functionality [48,49].

For instance, higher pyrolysis temperatures tend to increase aromaticity and microporosity, thereby enhancing adsorption capacity for hydrophobic contaminants. In contrast, milder conversion conditions may preserve functional groups that are beneficial for interactions with polar compounds [50]. These differences highlight the importance of tailoring conversion conditions to the requirements of specific applications.

In this context, the development of lignin-derived biochar can be viewed as a materials design problem in which controlling the conversion pathway allows the resulting structure and functionality to be tuned. However, establishing predictive relationships between lignin structure, processing conditions, and material properties remains a major challenge due to the intrinsic heterogeneity of lignin. Thus, lignin conversion should be understood not only as a transformation process but as a tunable design step for engineering application-specific biochar materials [51].

To summarize the relationships discussed above, the transformation of lignin into functional biochar materials can be understood as a sequential and interdependent process linking molecular structure, thermochemical conversion, and resulting physicochemical properties to final application performance.

As illustrated in Figure 2, lignin structure determines the pathways of thermochemical conversion, which in turn control the development of porosity, aromaticity, and surface functionality in lignin-derived biochar. These properties ultimately govern its performance in environmental remediation and controlled release systems.

Importantly, while thermochemical conversion defines the fundamental structure of lignin-derived biochar, additional modification strategies may be required to optimize performance for specific applications [10,52]. These post-conversion approaches, including activation and surface functionalization, are discussed in detail in the following section.

## 4. Functionalization of Lignin-Derived Biochar

The functionalization strategies discussed in this section can be interpreted within the structure–property–function framework illustrated in Figure 2. Functionalization strategies for lignin-derived biochar can be implemented either before, during, or after thermochemical conversion, depending on the desired material properties and application targets [53,54]. In addition to conventional post-treatment approaches applied to preformed biochars, several studies have demonstrated that direct activation or heteroatom incorporation into lignin precursors prior to carbonization can effectively tailor pore development, surface chemistry, and electronic properties of the resulting carbon materials [54,55,56]. These integrated approaches may combine carbonization and activation into a single processing step, potentially reducing energy consumption and simplifying process design compared with sequential post-treatment methods [57]. Consequently, functionalization should be understood as a broader material engineering strategy encompassing precursor-level modification, in situ transformation during thermochemical conversion, and post-synthesis treatments. These strategies are particularly relevant because the performance of pristine biochar, although often satisfactory, may still be limited by insufficient active-site density, restricted pore accessibility, or inadequate selectivity toward target molecules [27].

Functionalization is especially relevant in lignin-derived systems because the aromatic framework and residual oxygen-containing groups inherited from lignin provide a favorable platform for further chemical modification. Lignin-derived biochars already exhibit high carbon stability, pronounced aromatic character, and significant adsorption potential compared with biochars obtained from other lignocellulosic fractions, making them suitable candidates for engineered post-treatments [2,58].

### 4.1. Chemical Activation

Chemical activation is one of the most widely used post-treatment strategies for increasing surface area and developing microporosity. Activating agents such as potassium hydroxide (KOH), phosphoric acid (H_3_PO_4_), and zinc chloride (ZnCl_2_) promote pore development and generate highly porous carbon materials with enhanced adsorption capacity [10,59]. In lignin-derived biochars, this approach is particularly effective because the condensed aromatic skeleton formed during conversion can be selectively etched and reorganized without fully compromising structural integrity [60,61].

In several studies, activating agents such as KOH or H_3_PO_4_ have been directly mixed with raw lignin prior to carbonization, allowing simultaneous activation and pyrolysis in a single processing step. This strategy can enhance porosity development while reducing processing time and energy demand compared with sequential post-activation methods. In particular, H_3_PO_4_ activation has been associated with the development of mesoporosity and the preservation of oxygen-containing surface functionalities [54], whereas KOH activation typically promotes highly microporous structures with very high surface areas. For example, Feng et al. [60] impregnated lignin with KOH before thermal treatment and demonstrated that the activating agent promoted the formation of a highly developed porous network during carbonization, resulting in porous carbons with excellent electrochemical and adsorption properties. These results illustrate how precursor-level activation can directly influence pore development and surface chemistry during the carbonization process.

From an application perspective, chemical activation is especially useful when high surface area and abundant microporosity are required, as in the adsorption of organic pollutants or the immobilization of bioactive compounds. However, increased porosity must be balanced against possible losses in surface functionality and the risk of structural collapse under harsh treatment conditions. Thus, activation should be regarded not simply as a route to maximize surface area, but as a design tool for tuning the balance between accessibility, retention strength, and reactivity [49,62].

### 4.2. Heteroatom Doping

Heteroatom incorporation can be achieved either through post-treatment modification or by introducing nitrogen-, sulfur-, or phosphorus-containing precursors directly into the lignin matrix prior to thermochemical conversion. These heteroatoms can introduce additional active sites and modify the electronic properties of the carbon matrix, thereby improving adsorption performance and catalytic activity [63].

In lignin-derived biochar, heteroatom doping is particularly attractive because the pre-existing aromatic structure can stabilize the doped functionalities and facilitate their integration into the carbon matrix [64]. This type of modification may alter surface polarity, acid–base behavior, and charge distribution, thereby influencing interactions with ionic contaminants, aromatic molecules, and nutrient species [65,66]. Therefore, heteroatom doping provides a route beyond non-specific adsorption and toward more selective, application-oriented material design.

### 4.3. Metal Impregnation and Hybrid Functionalization

Metal impregnation is widely used to produce functional biochar composites. In this approach, metal nanoparticles or metal oxides, such as those based on iron, manganese, or magnesium, are introduced onto or into the biochar matrix, generating hybrid materials with enhanced adsorption capacity and catalytic properties [67]. These systems are particularly attractive because they can combine pollutant adsorption with catalytic degradation, thereby expanding the functional scope of lignin-derived biochar beyond passive sorption.

For lignin-derived biochar, this strategy is especially relevant because the oxygen-containing surface groups can serve as anchoring sites for metal species. In addition, the porous aromatic matrix provides stable support that can promote dispersion of the active phases and reduce aggregation. This hybridization approach is also promising for multifunctional agricultural systems in which adsorption, micronutrient retention, and controlled release may need to be integrated within a single material platform [68,69].

### 4.4. Surface Oxidation and Polarity Control

In addition to activation and impregnation, surface oxidation treatments can be applied to introduce additional oxygen-containing functional groups onto the biochar surface. Oxidation using agents such as nitric acid or hydrogen peroxide can improve hydrophilicity and surface reactivity, thereby enhancing interactions with polar contaminants and nutrient species [10,70].

This type of modification is particularly relevant when the target application depends not only on sorption capacity but also on controlled interaction strength. For example, in controlled release systems, excessively hydrophobic surfaces may retain active compounds too strongly, whereas oxidized surfaces may facilitate a more balanced adsorption–desorption behavior [71]. Consequently, surface oxidation provides a practical route for tuning polarity and interfacial behavior without fundamentally altering the carbon backbone of LDB [72].

### 4.5. Functionalization-Performance Relationships

Overall, the functionalization strategies discussed above provide effective tools for tailoring the properties of lignin-derived biochar and expanding its range of applications. In this context, functionalization should not be viewed as a simple enhancement step, but rather as a strategic design tool to tailor LDB performance toward specific applications.

By controlling surface chemistry, porosity, and structural accessibility, engineered LDB materials can be designed for specific uses such as emerging contaminant removal, catalysis, and controlled release systems [10,73].

However, a critical perspective is required, as enhanced functionality does not automatically translate into improved application performance. Excessive oxidation may reduce aromatic interactions, aggressive activation may compromise structural stability, and metal impregnation may raise concerns related to leaching or long-term environmental behavior [71]. Therefore, the rational design of functionalized LDB should focus not only on maximizing individual properties but also on achieving an appropriate balance between structure, reactivity, and application-specific performance [74].

Table 1 summarizes recent studies on lignin-derived biochar, highlighting the relationship between synthesis conditions, physicochemical properties, and environmental applications. These results demonstrate that activation methods and pyrolysis temperature play a key role in controlling surface area and pore structure, which directly influence the adsorption capacity of the resulting materials. Despite the wide variation in reported surface areas and adsorption capacities, direct comparison between studies remains challenging due to differences in lignin type, activation method, and experimental conditions. This highlights the need for standardized evaluation protocols.

Although adsorption capacities reported in the literature vary widely, several general trends can still be identified. Materials produced at higher pyrolysis temperatures and subjected to chemical activation typically exhibit larger surface areas and enhanced microporosity, which favor adsorption of hydrophobic organic contaminants. For example, KOH activation has been reported to increase the BET surface area of lignin-derived carbons to values exceeding 2000 m^2^ g^−1^ [60,75], whereas steam-activated materials commonly exhibit surface areas in the range of 500–800 m^2^ g^−1^ [76,77]. In contrast, oxidative treatments generally produce more moderate changes in porosity but substantially increase the abundance of oxygen-containing functional groups, enhancing interactions with polar compounds and metal ions. Similarly, nitrogen doping introduces basic surface functionalities that may improve adsorption selectivity toward specific contaminants, while metal impregnation can provide additional active sites for adsorption or catalytic reactions (Table 1). However, quantitative comparison among studies remains difficult because adsorption performance is strongly influenced by experimental variables such as pH, contaminant concentration, ionic strength, contact time, and normalization criteria.

**Table 1:** Lignin-derived biochar for environmental pollutant removal: influence of precursor, processing conditions, and material properties on adsorption performance.

Lignin Precursor	Pyrolysis/Synthesis Conditions	Activation/Modification	Key Properties	Dominant Mechanism	Application/Performance	Reference
Lignin (various)	Pyrolysis (600 °C)	ZnCl_2_ activation	Developed porosity; aromatic structure	π–π interactions, pore filling	Dye adsorption	[62]
Kraft lignin in black liquor	Pyrolysis (850 °C)	Self-activation + N-doping	Surface area: 1124 m^2^·g^−1^; porous structure	π–π interactions, electrostatic attraction	Methyl orange adsorption	[78]
Kraft lignin	Microwave pyrolysis	H_3_PO_4_ activation	Surface area: 635–1055 m^2^·g^−1^; micro/mesoporous	Pore filling, electrostatic interactions	Methylene blue and amoxicillin removal	[79]
Alkali lignin	Pyrolysis (800 °C)	Potassium tartrate activation	Surface area: 1911 m^2^·g^−1^; microporous	π–π interactions, pore filling	Methyl orange adsorption	[80]
Organosolv lignin	Pyrolysis (500–900 °C)	H_3_PO_4_ activation	>1000 m^2^·g^−1^; micro/mesoporous	Hydrophobic interactions, pore filling	Pharmaceutical adsorption	[81]
Lignosulfonate	Pyrolysis (850 °C)	CaCO_3_ templating	Surface area: 2944 m^2^·g^−1^; hierarchical porosity	Pore trapping, diffusion	Antibiotic adsorption	[82]
Biorefinery lignin	Pyrolysis (800 °C)	KHCO_3_ + melamine (N-doping)	Surface area: 3423 m^2^·g^−1^; highly porous	π–π interactions, electrostatic attraction	Methylene blue adsorption	[83]
Lignin	Pyrolysis (900 °C)	FeCl_3_/H_3_PO_4_/KOH activation	467–1389 m^2^·g^−1^; micro/mesoporous	Hydrophobic + electrostatic interactions	Nanoplastic adsorption	[84]
Lignin-based magnetic biochar	Hydrothermal (200 °C)	Fe_3_O_4_ modification	Magnetic, 152 m^2^·g^−1^	Electrostatic + redox interactions	Cr(VI) + dye removal	[85]
Organosolv lignin	NR	Fe_3_O_4_ nanoparticles	Functionalized surface	Adsorption + catalytic effects	Textile dye removal	[86]

NR: Not reported.

**Figure 3:**
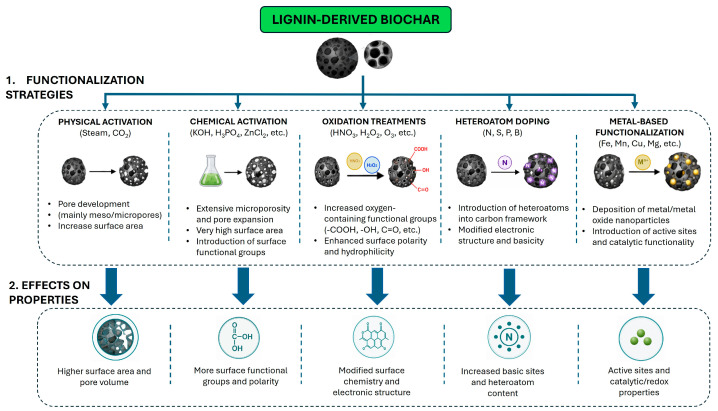
Main functionalization strategies of lignin-derived biochar and their effects on key material properties and potential applications.

To facilitate comparison among the different modification strategies discussed above, Figure 3 summarizes the main functionalization approaches applied to lignin-derived biochar and their effects on key material properties. As illustrated, each modification pathway selectively enhances characteristics such as porosity, surface functionality, electronic properties, or active-site availability, thereby expanding the potential applications of lignin-derived biochar in adsorption, catalysis, environmental remediation, and controlled release systems.

## 5. Applications of Lignin-Derived Biochar

Building upon the structure-property relationships established in previous sections, lignin-derived biochar can be applied in two main domains: (i) adsorption of emerging contaminants and (ii) controlled release systems. These correspond to two complementary operational regimes, in which the same physicochemical properties govern either retention-dominated or release-controlled behavior. These domains are directly linked to the structure–property–function relationships illustrated in Figure 2, which provide a unifying framework for interpreting both adsorption and controlled release behaviors. In particular, the high aromaticity inherited from lignin, combined with tunable porosity and diverse surface functional groups, enables the development of carbon materials with strong and versatile interactions with a wide range of organic molecules and ionic species [49,53]. This dual functionality underscores the need to interpret lignin-derived biochar systems not as static adsorbents, but as dynamic platforms in which adsorption and release represent two interconnected and tunable processes.

The performance of lignin-derived biochar in adsorption applications is commonly evaluated through kinetic, equilibrium, and thermodynamic analyses [14,87,88]. Pseudo-first-order and pseudo-second-order kinetic models are frequently employed to describe adsorption rates and identify potential rate-controlling mechanisms. Likewise, adsorption isotherms such as the Langmuir and Freundlich models are widely used to assess adsorption capacity, surface heterogeneity, and adsorbate–adsorbent interactions. Thermodynamic parameters, including Gibbs free energy (ΔG°), enthalpy (ΔH°), and entropy (ΔS°), further provide insight into the spontaneity and energetic characteristics of the adsorption process. Together, these approaches contribute to a more comprehensive understanding of contaminant removal mechanisms and facilitate comparison among lignin-derived biochars prepared under different conditions.

From a biorefinery perspective, these applications represent a direct valorization pathway for technical lignins, transforming a traditionally underutilized by-product into high-value functional materials. The ability to tailor LDB properties through controlled thermochemical conversion and post-functionalization further reinforces its role as a platform material within integrated lignocellulosic biorefineries. Importantly, both applications are governed by the same structure–property–function relationships, although they differ in their operational objectives, namely maximizing retention in adsorption systems and enabling regulated release in delivery applications.

### 5.1. Adsorption of Emerging Contaminants

Emerging contaminants, including pharmaceuticals, personal care products, endocrine-disrupting compounds, microplastics, and per- and polyfluoroalkyl substances (PFAS), are increasingly detected in aquatic environments due to their widespread use and incomplete removal in conventional treatment systems [89]. In this context, lignin-derived biochar has attracted increasing attention as a sustainable adsorbent because of its renewable origin and tunable physicochemical properties [53,54].

**Figure 4:**
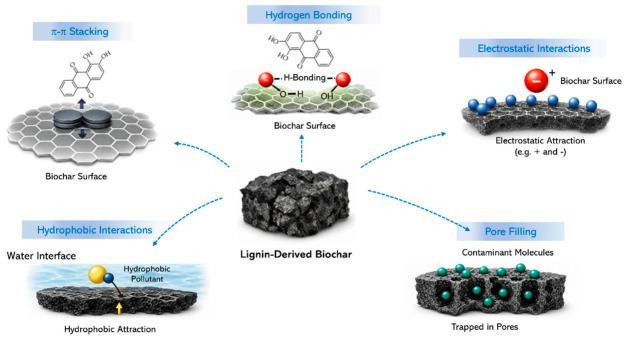
Main mechanisms involved in the adsorption of emerging contaminants on lignin-derived biochar, including π–π stacking, hydrogen bonding, electrostatic interactions, pore filling, and hydrophobic interactions. These mechanisms are governed by the aromatic structure, surface functional groups, and porosity of LDB.

Adsorption performance is governed by the interplay between the aromatic carbon framework, pore structure, and surface chemistry of LDB. The main adsorption mechanisms involved in the interaction between lignin-derived biochar and emerging contaminants are summarized in Figure 4. These include π–π stacking interactions associated with the aromatic domains inherited from lignin, hydrogen bonding mediated by oxygen-containing functional groups, electrostatic attraction between charged species and surface sites, pore-filling effects within the porous structure, and hydrophobic interactions at the biochar–water interface. The relative contribution of each mechanism depends on both the physicochemical properties of the contaminant and the structural characteristics of LDB. These mechanisms (Figure 4) underpin the adsorption behavior observed across the different contaminant classes discussed below.

#### 5.1.1. Pharmaceuticals and Antibiotics

Pharmaceutical compounds represent one of the most prevalent classes of emerging contaminants due to their continuous release into aquatic environments and resistance to degradation [90]. Lignin-derived carbon materials have demonstrated strong adsorption capacity for a wide range of pharmaceutical molecules.

Popovic et al. [91] reported efficient diclofenac removal using amino-functionalized lignin microspheres, in which adsorption was primarily governed by electrostatic interactions and hydrogen bonding. Similarly, lignin-derived hierarchical porous carbons exhibit high affinity for carbamazepine because of their developed surface area and interconnected pore structures, which facilitate diffusion and adsorption within the carbon matrix [82].

Antibiotic removal has also been extensively studied. Xiang et al. [16] reported tetracycline adsorption capacities of approximately 31 mg g^−1^ using lignin-modified biochar, whereas significantly higher capacities (>400 mg g^−1^) were achieved with KOH-activated systems due to enhanced microporosity and surface functionality [92]. These results highlight the critical role of activation processes in tailoring lignin-derived structures for improved adsorption performance.

#### 5.1.2. Personal Care Products and Endocrine-Disrupting Compounds

Personal care products (PCPs) and endocrine-disrupting compounds (EDCs) are typically aromatic molecules, making them particularly susceptible to adsorption onto lignin-derived carbon materials. The aromatic structure inherited from lignin plays a decisive role in promoting π–π stacking and hydrophobic interactions.

For instance, Tong et al. [93] and Cho et al. [94] demonstrated that triclosan adsorption is strongly influenced by pyrolysis temperature, surface area, and functional group distribution, with reported capacities of up to ~49 mg g^−1^. Similarly, lignin-derived activated carbons exhibit high adsorption capacity for bisphenol A (BPA), reaching approximately 220–260 mg g^−1^ [54,95], with performance closely linked to microporosity and surface chemistry.

These findings suggest that the adsorption of aromatic contaminants is fundamentally controlled by the graphitic domains formed during lignin carbonization, highlighting the importance of lignin-derived structural features.

#### 5.1.3. Microplastics and Nanoplastics

The removal of microplastics and nanoplastics represents an emerging application area for LDB materials. Their hydrophobic nature and persistence in aquatic systems make them difficult to remove using conventional technologies.

Recent studies have shown that lignin-derived activated carbons with high surface area (~1300 m^2^ g^−1^) can effectively adsorb polystyrene nanoplastics, primarily through hydrophobic interactions and physical trapping within micropores [84]. In this context, pore structure and surface roughness are key parameters governing adsorption performance.

Despite promising results, this research area remains relatively underdeveloped, particularly for biochars derived exclusively from isolated lignin, highlighting a relevant gap in the current literature. In addition to pore-filling effects, the adsorption of microplastics and nanoplastics by lignin-derived biochar may be influenced by hydrophobic interactions, π–π interactions between aromatic carbon structures and polymer surfaces, and electrostatic forces depending on the surface chemistry of both the adsorbent and the plastic particles [96,97]. Compared with commercial activated carbons, LDB materials generally exhibit lower adsorption capacities due to their less developed porosity and surface area. However, they offer important advantages related to renewable feedstock utilization, lower production costs, and integration within biorefinery-based valorization schemes [10,50,53]. Therefore, further studies are needed to optimize LDB physicochemical properties and to establish direct performance comparisons with commercial activated carbons under standardized experimental conditions. In particular, future work should evaluate adsorption performance under environmentally relevant conditions and assess the influence of microplastic characteristics, including polymer type, particle size, surface aging, and the presence of co-contaminants.

#### 5.1.4. PFAS

PFAS represent one of the most challenging classes of emerging contaminants due to their high chemical stability and persistence. Compared with other contaminant classes, studies focusing on lignin-derived biochar for PFAS removal remain limited.

Mel et al. [15] demonstrated that kraft lignin can adsorb PFAS compounds, with sorption behavior strongly influenced by chain length and functional group chemistry. Roman et al. [98] further showed that carbonization of lignin significantly enhances PFAS adsorption capacity by increasing surface area and pore accessibility. Moreover, lignin-derived activated carbons have been reported to achieve removal efficiencies exceeding 99% for compounds such as perfluorooctanoic acid (PFOA) and perfluorooctane sulfonate (PFOS) under optimized conditions [99].

However, despite these advances, LDB-based systems still require optimization to match the performance of commercial activated carbons, particularly in terms of microporosity and surface chemistry.

Overall, adsorption in lignin-derived biochar systems is governed by a combination of aromaticity, surface functionality, and pore architecture. These properties, inherited from lignin structure and modulated through thermochemical conversion, determine the relative contribution of different interaction mechanisms, including π–π stacking, hydrogen bonding, electrostatic attraction, pore filling, and hydrophobic interactions (Figure 4). Together, these mechanisms explain the high affinity of LDB for a wide range of emerging contaminants and highlight its versatility as a sustainable adsorbent.

However, adsorption performance is often reported under idealized laboratory conditions, and real-world applicability, particularly in complex environmental matrices, remains insufficiently validated. Most studies rely on simplified systems, such as single-solute solutions under controlled pH and short-term batch conditions, which may significantly overestimate performance compared to real environments. Furthermore, the lack of standardized testing protocols, including differences in initial concentrations, contact times, and normalization approaches, hinders direct comparison across studies. Addressing these limitations is essential to advance lignin-derived biochar from laboratory-scale demonstrations to practical environmental applications. Future studies should prioritize continuous-flow systems, multi-contaminant matrices, and pilot-scale validation to better assess real-world applicability.

**Figure 5:**
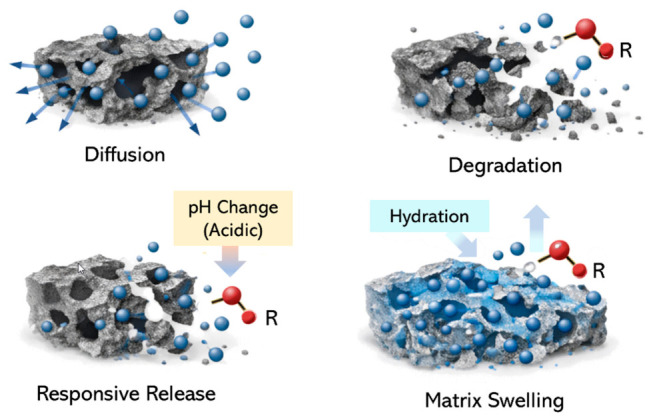
Main mechanisms involved in the controlled release of active compounds from lignin-derived biochar (LDB), including diffusion, matrix degradation, stimulus-responsive release, and swelling-induced release. In LDB systems, these mechanisms are strongly influenced by the aromatic carbon framework, pore structure, and surface functional groups inherited from lignin, which collectively determine release kinetics and environmental responsiveness.

From a valorization perspective, the use of LDB as an adsorbent represents a high-value application for lignin streams within biorefineries, contributing to environmental remediation while improving carbon utilization efficiency. Importantly, adsorption and controlled release are not independent phenomena but complementary manifestations of the same structure-property-function relationships. This conceptual continuity provides the basis for the controlled release systems discussed in the following section. Importantly, the mechanisms described for adsorption (Figure 4) form the basis for controlled release behavior, as the same interactions governing retention must be finely tuned to enable regulated desorption in controlled release systems (Figure 5).

### 5.2. Controlled Release Systems Based on Lignin-Derived Biochar

The adsorption properties of lignin-derived biochar discussed in the previous section highlight the strong affinity of these carbonaceous materials for a wide range of organic molecules and ionic species [50,73]. The same physicochemical characteristics that make lignin-derived biochar effective for contaminant removal, such as high surface area, developed pore structure, aromatic carbon domains, and abundant surface functional groups, also make these materials highly suitable as carriers for controlled release systems [9,10].

It should be noted that, while this section focuses on lignin-derived biochar, the current literature is still dominated by lignin-based systems, particularly in pesticide delivery applications [100,101,102]. In this context, the term lignin-derived carbon materials is used to encompass both biochar and closely related carbonaceous structures obtained from lignin. At present, fully biochar-based controlled release systems derived exclusively from isolated lignin remain comparatively underdeveloped. Therefore, this section also discusses lignin-derived porous carbons and lignin-based hybrid carriers to provide a comprehensive overview of the current state of the field. This distinction is important because many controlled release strategies still rely on lignin matrices or lignin-based composites rather than standalone lignin-derived biochar systems.

In adsorption applications, biochar acts as a sorbent that immobilizes contaminants from aqueous environments. In contrast, in controlled release systems, the same adsorption capacity is exploited to temporarily retain nutrients, pesticides, or other bioactive compounds within the porous structure of the material and subsequently release them in a gradual and regulated manner. This dual functionality further highlights the conceptual link between adsorption and controlled release in LDB systems [10,73].

Consequently, increasing research efforts have been devoted to the development of controlled release systems based on lignin-derived biochar, particularly in the context of sustainable agriculture and nutrient management [9]. These systems aim to improve agrochemical efficiency while minimizing environmental losses such as leaching, volatilization, and degradation [103].

To provide a structured overview, Table 2 compiles representative experimental studies on lignin-derived carbon materials and lignin-based systems used in controlled release applications, including precursor type, synthesis and modification strategies, physicochemical properties, dominant release mechanisms, and performance metrics. Given the limited number of studies focusing exclusively on lignin-derived biochar, representative lignin-based carriers and hybrid systems are also included to provide a comprehensive overview of current controlled release strategies derived from lignin.

Although the controlled release systems summarized in Table 2 differ considerably in composition, target compounds, and evaluation methodologies, several common trends can be identified. Materials with highly developed porosity generally exhibit greater loading capacity and stronger retention of active compounds, whereas lignin-based coatings and hybrid matrices primarily regulate release through diffusion barriers and hydrophobic effects. Furthermore, release kinetics are strongly influenced by the balance between adsorption strength and molecular mobility within the carrier matrix. However, direct quantitative comparison among studies remains challenging because performance is reported using different release metrics, experimental conditions, and target compounds.

The collected studies illustrate how controlled release behavior emerges from the interplay between surface chemistry, porosity, and matrix design, which together regulate adsorption strength and diffusion pathways, as conceptually represented in Figure 5, where the main release mechanisms are illustrated.

Importantly, Table 1 and Table 2 should be interpreted as complementary datasets representing two operational regimes of lignin-derived biochar (LDB) systems: retention-dominated behavior in contaminant adsorption (Table 1) and balanced retention-release behavior in controlled release systems (Table 2). While adsorption applications prioritize strong and often irreversible interactions, controlled release systems require a fine-tuned balance between affinity and mobility to ensure sustained and effective delivery of active compounds [10,50].

Notably, the studies compiled in Table 2 also reveal that, whereas lignin-derived porous carbons are more frequently applied in nutrient controlled release systems, pesticide-related applications are still largely dominated by lignin-based carriers and composite formulations rather than standalone biochar matrices [107,108,110]. This distinction underscores current research trends and highlights opportunities for further development of LDB-specific platforms in controlled release technologies.

#### 5.2.1. Controlled Release Fertilizers

Lignin-derived biochar has been increasingly explored as a carrier material for controlled release fertilizers due to its porous structure, high surface area, and tunable surface chemistry [9,10]. These properties enable the adsorption and retention of nutrients within the biochar matrix, followed by their gradual release into the soil solution.

In nitrogen-based systems, lignin-derived carbon materials and lignin-biochar composites have demonstrated the ability to reduce nutrient losses through leaching while extending nutrient availability over time [104,105]. The incorporation of porous carbon structures enhances nutrient retention via physical adsorption and pore confinement, whereas surface functional groups contribute to interactions with ionic species such as ammonium and nitrate [50].

Similarly, lignin-based coatings and composites have been applied to urea fertilizers to regulate nutrient release kinetics. In these systems, diffusion barriers created by lignin-derived matrices or hybrid materials control water penetration and nutrient dissolution, resulting in prolonged release profiles and improved nutrient use efficiency [106]. These diffusion- and barrier-governed processes are among the mechanisms summarized in Figure 5.

Beyond diffusion-controlled release, some lignin-derived systems exhibit swelling-induced and stimulus-responsive release behaviors [111,112,113,114]. In swelling-induced systems, water uptake by the carrier matrix promotes structural expansion, increasing pore accessibility and facilitating the diffusion of encapsulated nutrients. The extent of swelling depends on factors such as matrix composition, porosity, and the presence of hydrophilic functional groups. More advanced lignin-based formulations can also respond to environmental stimuli such as pH, temperature, moisture, or enzymatic activity, allowing nutrient release rates to adapt to local conditions. For example, Jiang et al. [111] developed lignin–polylactic acid microspheres capable of responding to environmental pH changes, resulting in more controlled agrochemical release and prolonged retention of the active compound. Similarly, Lin et al. [114] reported lignin-coated mesoporous nanocarriers exhibiting dual pH- and temperature-responsive behavior, in which alkaline conditions and elevated temperatures accelerated the release of the encapsulated pesticide through the progressive disruption of intermolecular interactions within the lignin coating. These responsive mechanisms have attracted increasing attention because they can synchronize nutrient availability with environmental conditions and plant demand, potentially improving nutrient use efficiency while reducing losses associated with premature release. Nevertheless, further studies are still required to evaluate the long-term performance and scalability of these systems under field conditions.

Overall, the performance of lignin-derived systems in fertilizer delivery is governed by the balance between adsorption capacity and diffusion limitations, which can be tuned through pyrolysis conditions, activation strategies, and composite design [9].

#### 5.2.2. Controlled Release of Pesticides

In addition to fertilizers, lignin-derived materials have also been investigated as carriers for the controlled release of pesticides [101]. Conventional pesticide formulations often suffer from rapid degradation, volatilization, or leaching after application, which reduces their effectiveness and increases the risk of environmental contamination [103].

##### Herbicides

Herbicides are widely used to control weed growth in agricultural systems, but their excessive application can lead to soil and water contamination. Lignin-derived materials have been explored as delivery platforms to regulate herbicide release and reduce environmental losses [100,102].

The interaction between herbicides and lignin-derived matrices is typically governed by π–π interactions, hydrogen bonding, and hydrophobic partitioning, depending on the chemical structure of the active compound and the surface properties of the carrier [50,73]. These interactions enable the temporary retention of herbicides within the material, followed by a controlled release driven by diffusion and desorption processes, as illustrated in Figure 5.

##### Insecticides and Other Pesticides

Controlled release systems based on lignin-derived materials have also been developed for insecticides and other bioactive compounds. In many cases, lignin-based carriers, microcapsules, and hybrid systems are employed to enhance stability and regulate release kinetics [107,108,110].

Encapsulation strategies provide additional protection against photodegradation and environmental degradation, while diffusion-controlled mechanisms ensure sustained release over time. These systems often combine adsorption within the lignin matrix with barrier-controlled release, resulting in improved persistence and reduced losses [109]. These observations are consistent with the broader trends summarized in Table 2, where pesticide controlled release systems are predominantly based on lignin-derived carriers and composite formulations rather than standalone biochar matrices.

Despite these promising developments, studies specifically focusing on lignin-derived biochar as standalone carriers for pesticide delivery remain scarce, highlighting a critical gap in the current literature.

## 6. Challenges and Future Perspectives

Despite the significant progress achieved in the valorization of lignin into functional carbon materials, the development of lignin-derived biochar for environmental remediation and controlled release systems remains at a relatively early stage within the broader context of biorefinery integration. While numerous studies have demonstrated the potential of LDB as a multifunctional material, several interconnected challenges must be addressed to enable its transition from laboratory-scale research to scalable and application-oriented technologies.

### 6.1. Lignin Heterogeneity and Feedstock Variability

One of the most critical limitations in the development of LDB materials lies in the intrinsic structural heterogeneity of lignin. Unlike synthetic polymers, lignin exhibits a highly irregular and source-dependent architecture composed of varying proportions of H, G, and S units, as well as diverse interunit linkages and functional groups.

This variability is further amplified by industrial extraction processes, leading to different types of technical lignin (e.g., kraft, organosolv, and lignosulfonates), each with distinct physicochemical characteristics. As a result, the lignin feedstock strongly influences thermochemical conversion pathways, carbon yield, pore development, and surface functionality in the resulting biochar [1,31].

Consequently, establishing robust and predictive structure–property–function relationships remains a major challenge. Current studies often rely on empirical optimization, with limited ability to correlate lignin molecular structure with final material performance. Addressing this limitation is essential for the rational design of lignin-derived biochar tailored to specific applications.

### 6.2. Limited Understanding of Molecular-Level Mechanisms

Closely related to feedstock variability is the limited understanding of the molecular mechanisms governing lignin transformation during thermochemical conversion and its impact on LDB functionality. Although these mechanisms are commonly reported (Figure 4), their relative contributions and synergistic effects remain poorly understood, particularly for biochars derived exclusively from lignin, thereby limiting the development of predictive structure–property–function relationships.

Although it is well established that lignin aromaticity contributes to the formation of condensed carbon structures, the role of specific functional groups, monolignol composition, and linkage distribution in determining porosity, surface chemistry, and reactivity is not yet fully understood [39,40]. This knowledge gap restricts the ability to design LDB materials with targeted adsorption or controlled-release properties.

Advanced analytical techniques such as solid-state NMR, X-ray photoelectron spectroscopy (XPS), and high-resolution electron microscopy, combined with computational modeling approaches, are expected to play a central role in elucidating these relationships. Recent studies have increasingly combined experimental characterization with molecular simulations and density functional theory (DFT) calculations to better understand adsorption mechanisms and heteroatom effects in biochar systems [61,65,115]. These approaches may help establish predictive relationships between lignin chemistry, thermochemical conversion pathways, and adsorption behavior, thereby supporting rational LDB design. Bridging this gap will enable a shift from empirical material development toward predictive and design-oriented strategies.

### 6.3. Process Optimization and Scalability

From a technological perspective, the scalability of lignin-to-biochar conversion remains a critical challenge. Thermochemical processes such as pyrolysis and hydrothermal carbonization offer flexibility in tailoring LDB properties; however, their performance is highly sensitive to process parameters, including temperature, residence time, heating rate, and activation conditions [9].

While laboratory studies have demonstrated the feasibility of tuning porosity and surface chemistry, scaling these processes to industrial levels involves significant challenges related to energy consumption, process control, and reproducibility. In addition, the integration of activation and functionalization may further increase process complexity and cost [116].

Within the context of lignocellulosic biorefineries, the efficient integration of LDB production with upstream lignin fractionation and downstream applications is essential. Developing continuous, energy-efficient, and economically viable processes will be a key factor in determining the industrial feasibility of lignin-derived biochar technologies.

### 6.4. Balancing Adsorption Strength and Release Performance

In both environmental remediation and controlled-release applications, LDB performance is governed by a delicate balance between adsorption strength and desorption behavior. While high aromaticity and developed microporosity enhance the adsorption of contaminants and agrochemicals, excessively strong interactions may hinder desorption and reduce bioavailability.

This trade-off is particularly critical in controlled-release systems, where overly strong retention of nutrients or pesticides can limit their availability to plants or target organisms [117]. Conversely, insufficient interaction strength may lead to rapid release and reduced efficiency. As illustrated in Figure 5, controlled-release behavior is governed by multiple concurrent mechanisms, including diffusion, matrix degradation, and environmental responsiveness, which must be carefully balanced to achieve optimal performance.

Achieving optimal performance therefore requires precise control of surface chemistry, pore structure, and functional group distribution. This highlights the importance of rational material design strategies that consider both adsorption capacity and release kinetics as interdependent parameters.

### 6.5. Need for Lignin-Specific Studies

A significant limitation in the current literature is the predominance of studies based on biochars derived from whole lignocellulosic biomass rather than from isolated lignin. While these materials provide valuable insights, they do not fully capture the unique structural features and reactivity of lignin-derived systems.

Given that lignin plays a dominant role in determining aromaticity, pore development, and surface functionality, LDB materials are expected to exhibit distinct behavior compared with conventional biochars. However, systematic studies focusing exclusively on lignin-derived biochar remain limited.

This represents a critical research gap, particularly in applications such as pesticide delivery and PFAS adsorption, where lignin-specific interactions may offer unique advantages. Future research should therefore prioritize comparative studies that isolate the role of lignin in determining material performance.

### 6.6. Functionalization and Hybrid Material Design

Functionalization and hybridization strategies offer promising routes for overcoming current limitations in LDB performance. As discussed in Section 4, approaches such as heteroatom doping, metal impregnation, and polymer coating can significantly modify surface chemistry, porosity, and reactivity.

These modifications enable the development of multifunctional materials capable of combining adsorption, catalysis, and controlled release within a single platform. For example, metal-modified LDB can enhance contaminant removal through catalytic degradation, whereas polymer-biochar composites can improve control over release kinetics in agricultural systems [67,69].

However, these strategies also introduce new challenges, including increased synthesis complexity, potential environmental risks associated with metal leaching, and higher production costs. Therefore, the design of functionalized LDB materials should focus on achieving an optimal balance between performance, stability, and sustainability.

### 6.7. Environmental and Economic Assessment

For LDB technologies to be successfully implemented at scale, their environmental and economic performance must be rigorously evaluated. Life cycle assessment (LCA) and techno-economic analysis (TEA) are essential tools for assessing the sustainability of lignin-derived biochar production and application [116].

These evaluations should consider not only energy inputs and production costs but also long-term environmental impacts, including soil persistence, carbon sequestration potential, ecotoxicity, and the fate of adsorbed contaminants or released agrochemicals.

In addition, the development of standardized protocols for evaluating adsorption performance, release kinetics, and environmental safety is necessary to ensure comparability among studies and to facilitate regulatory acceptance.

### 6.8. Future Research Directions

Looking forward, the development of lignin-derived biochar as a key material within sustainable biorefineries will depend on several priority research areas, including the establishment of predictive structure–property–function relationships linking lignin chemistry to biochar performance, the development of scalable and energy-efficient thermochemical conversion processes, the design of lignin-specific materials for targeted applications such as PFAS removal and pesticide delivery, the advancement of multifunctional and hybrid systems that integrate adsorption and controlled release, and the validation of performance under realistic environmental and field conditions. From a broader perspective, LDB represents a strategic bridge between lignin chemistry and functional material design. Its ability to transform low-value lignin streams into high-performance materials aligns strongly with the principles of the circular bioeconomy and sustainable resource utilization [51].

## 7. Conclusions and Integration in the Circular Bioeconomy

Lignin, as the most abundant renewable aromatic polymer in nature, represents a key yet historically underutilized component of lignocellulosic biomass within modern biorefineries. Traditionally valorized through low-value energy recovery, lignin is increasingly recognized as a strategic feedstock for producing advanced carbon materials. Among these, lignin-derived biochar (LDB) has emerged as a versatile platform due to its high carbon content, tunable physicochemical properties, and intrinsic aromatic structure.

As summarized in Figure 2, the performance of LDB is governed by interconnected structure–property–function relationships, where lignin molecular structure, thermochemical conversion pathways, and the resulting material properties collectively determine its behavior in environmental and agricultural applications.

This review has highlighted that the transformation of lignin into biochar through thermochemical processes such as pyrolysis and hydrothermal carbonization enables the generation of functional carbon materials with controllable porosity, surface chemistry, and structural stability. Importantly, these properties are not arbitrary but are directly governed by the molecular structure of lignin and the conditions of its conversion, reinforcing the importance of establishing clear structure–property–function relationships for rational material design.

In environmental applications, LDB shows strong potential as a sustainable adsorbent for the removal of emerging contaminants, including pharmaceuticals, antibiotics, endocrine-disrupting compounds, microplastics, and PFAS. The effectiveness of these materials arises from the combined contribution of aromatic carbon domains, developed pore structures, and surface functional groups, which enable multiple interaction mechanisms such as π–π stacking, hydrogen bonding, electrostatic attraction, and pore-filling processes. These findings confirm that lignin-derived carbon materials can serve as efficient and renewable alternatives to conventional adsorbents in water treatment systems.

In parallel, LDB has shown considerable promise as a carrier in controlled release systems for agricultural applications. Its porous structure and surface chemistry allow the adsorption and gradual release of nutrients, pesticides, and micronutrients, contributing to improved agrochemical use efficiency and reduced environmental losses. Lignin-derived biochar offers advantages in terms of chemical stability, long-term persistence in soil, and tunability of release kinetics through material design and functionalization strategies. While adsorption processes (Figure 4) rely on maximizing interaction strength, controlled release systems (Figure 5) require a balance between retention and desorption.

From a biorefinery perspective, these applications represent a direct and high-value valorization pathway for lignin streams. By converting technical lignins into functional materials with environmental and agricultural relevance, LDB contributes to improved carbon utilization, reduced waste generation, and the development of integrated and circular processing schemes. Moreover, the multifunctionality of LDB, combining adsorption, controlled release, and potential catalytic activity, positions it as a key material for addressing interconnected challenges in water treatment and sustainable agriculture.

However, despite these advances, several challenges must be addressed to fully realize the potential of lignin-derived biochar. These include the intrinsic heterogeneity of lignin, the limited understanding of molecular-level transformation mechanisms, and the need for scalable and economically viable production processes. In addition, further research is required to optimize the balance between adsorption strength and release efficiency, particularly in controlled release applications, and to validate material performance under realistic environmental conditions.

A critical research gap lies in the limited number of studies focusing specifically on biochars derived exclusively from isolated lignin. Most existing work is based on lignocellulosic biomass, which obscures the unique contributions of lignin to material properties and performance. Addressing this gap will be essential for advancing lignin-specific design strategies and fully exploiting the potential of lignin-derived systems.

Looking forward, the development of LDB within biorefinery frameworks will require a shift toward more integrated and design-oriented approaches. This includes the establishment of predictive relationships linking lignin structure to material performance, the optimization of thermochemical conversion and functionalization processes, and the development of hybrid materials tailored for specific applications. In parallel, comprehensive life cycle and techno-economic assessments will be necessary to ensure the sustainability and feasibility of these technologies on a scale.

In conclusion, lignin-derived biochar represents a strategic bridge between lignin chemistry and functional material design. By enabling the transformation of an abundant and underutilized biopolymer into high-performance carbon materials, LDB aligns closely with the principles of circular bioeconomy.

Future advances will depend on the development of predictive structure–property–function relationships, scalable conversion processes, and application-driven material design strategies. With these advances, LDB has the potential to become a cornerstone technology in next generation biorefineries supporting more sustainable, efficient, and integrated biomass utilization, and positioning lignin-derived biochar as a key material in next-generation biorefineries.

## Figures and Tables

**Table 2:** Studies on lignin-derived porous carbon, lignin/biochar hybrid coatings, and lignin-based carriers for controlled release of fertilizers and pesticides.

Lignin Precursor	Pyrolysis/Synthesis Conditions	Activation/Modification	Key Properties	Dominant Mechanism	Application/Performance	Reference
Industrial alkaline lignin (IAL)	Hydrothermal carbonization/activation; optimum synthesis reported at 220 °C	Porous carbon from IAL + urea, extrusion granulation	Specific surface area 1923.51–1935.5 m^2^·g^−1^; pore volume 0.82 cm^3^·g^−1^	Nutrient adsorption in porous carbon + diffusion-limited release	Slow-release N fertilizer; soil-column tests showed lower cumulative leaching of NH_4_^+^-N, NO_3_^−^-N and total N than conventional urea	[104]
Wood-derived lignin + activated biochar	Bio-coating material prepared from wood waste; applied as coating on urea fertilizer	Palmitoyl-chloride-grafted lignin (PCL) + activated biochar (ABC)	Hydrophobic coating; water contact angle 90.72°; ABC with high surface area and hierarchical porosity	Hydrophobic barrier + pore-regulated ion transport	Coated slow-release urea; nitrogen release persistence for 40 days in soil; also reduced Cr accumulation in rice roots in hydroponics	[105]
Lignosulfonate/lignin sulfonate	Coating process for urea (not pyrolysis-based; lignin coating, not LDB)	Acetylation with decanoyl chloride to reduce hydrophilicity	Biodegradable coating membrane; diffusion coefficient modeled for N release	Diffusion-controlled release across lignin-derived coating	Controlled-release urea fertilizer; study demonstrates delayed nutrient transfer and models effective diffusion coefficient through the coating	[106]
Sodium lignosulfonate	High-speed emulsification + ultrasonic dispersion; crosslinking with p-phenylenediamine diazonium salt	Nano-delivery formulation	Particle size 80–150 nm; UV-shielding behavior	Encapsulation + pH-responsive diffusion/release	Avermectin (AVM) nanoformulation; anti-photolysis stability reported as 3–4× that of free AVM, with pH-responsive controlled release	[107]
Sodium lignosulfonate (self-assembled lignin microspheres)	Self-assembly of SL-CTAB colloidal spheres (not pyrolysis-based; lignin carrier, not LDB)	Surfactant-assisted microsphere formation	Uniform colloidal spheres; AVM encapsulation with high loading reported in abstract/snippet	Encapsulation + UV protection + sustained release	AVM carrier; lignin microspheres protect AVM against photodegradation and act as a controlled/sustained-release shell	[108]
Lignosulfonate granules	Enzymatic granulation using laccase (not pyrolysis-based; lignin carrier, not LDB)	100% lignosulfonate-based biodegradable solid carrier	Granular biobased carrier	Matrix dissolution + diffusion-controlled herbicide release	Dicamba delivery; complete release after 48 h according to HPLC analysis	[109]
Lignin particle-stabilized microcapsules	Pickering emulsion template + interfacial polymerization (not pyrolysis-based; lignin carrier, not LDB)	Lignin-polyurea microcapsules	Spherical capsules with double-layer shell; mean diameter 10–100 μm	Barrier-controlled sustained release + photoprotection	AVM carrier with anti-photolysis and sustained-release performance	[110]

## Data Availability

No new data were created or analyzed in this study. Data sharing is not applicable to this article.
